# Supplementary material for: Modeling dynamic oxygen permeability as a mechanism to mitigate oxygen-induced stresses on photosynthesis and N2 fixation in marine Trichodesmium
Source: Microbiol Spectr. 2025 Aug 13;13(10):e00453-25. doi: 10.1128/spectrum.00453-25 (PMC12502677; doi:10.1128/spectrum.00453-25)
Supplement: Supplemental material — Supplemental methods, Tables S1 to S4, and Fig. S1 to S7. [file spectrum.00453-25-s0001.pdf]

Modeling dynamic oxygen permeability as a mechanism to mitigate oxygen-induced stresses on photosynthesis and N_2 fixation in marine *Trichodesmium*

Weicheng Luo[1, 2]*, Keisuke Inomura[3], Ondřej Prášil[2], Meri Eichner[2], Ya-Wei Luo[1]*

[1]State Key Laboratory of Marine Environmental Science and College of Ocean and Earth Sciences, Xiamen University, Xiamen 361102, China.

[2]Centre Algatech, Institute of Microbiology of the Czech Academy of Sciences, Třeboň 37901, Czech Republic.

[3]Graduate School of Oceanography, University of Rhode Island, Narragansett, RI 02882, USA.

*Correspondence: weicheng123@stu.xmu.edu.cn; ywluo@xmu.edu.cn

SUPPLEMENTARY METHODS: Full model description

1. Photosynthetic pathways

Linear photosynthetic electron transfer (LPET) and alternative electron transfer (AET) are simulated in our model (Fig. 1A). Because (Mehler reaction)-mediated AET is the dominant AET in *Trichodesmium* (1), other AET (e.g., the cyclic electron transfer around photosystem I, the AET mediated by midstream oxidase with photosystem II, and the AET from photosystem II to the respiratory terminal oxidase respiratory terminal oxidase) (2), are not considered.

Both LPET and AET generate a proton gradient of 12 H^+ per 8 photons (3). By assuming that 3 ATP are produced by the thylakoid ATP synthase with a proton gradient of 14 H^+, for 4 electrons through PET, LPET produces 2.6 ATP, with 2 NADPH and 1 O_2, while AET only produces 2.6 ATP (2-4).

The total PET rate [V_{PET}, mol electron (mol C)$^{-1}$ s^{-1}] is regulated by light intensity (I, μmol m^{-2} s^{-1}) and the Fe quota in photosystems [Fe_{PS}, μmol Fe (mol C)$^{-1}$], and V_{PET} is inhibited by respiratory protection (RP) [V_{RP}, mol C (mol C)$^{-1}$ s^{-1}, described later] (5).

$$V_{PET} = v_{PET}^{max} \cdot \frac{Fe_{PS}}{Fe_{PS} + k_{Fe}^{PS}} \cdot (1 - e^{-\alpha_I \cdot I}) \cdot e^{-\beta \cdot V_{RP}}, \tag{S1}$$

where v_{PET}^{max} [mol electron (mol C)$^{-1}$ s^{-1}] is the maximal rate of PET, k_{Fe}^{PS} [μmol Fe (mol C)$^{-1}$] is the half-saturating coefficient of Fe_{PS} for PET, α_I (μmol^{-1} m^2 s) is the initial slope of PET versus light curve, and β [mol C (mol C)$^{-1}$ s] represents the degree of the inhibition effect from RP on PET. Note that in fixed-Fe and dynamic-Fe cases, the light intensity is diurnally variant with time, following the sine function during a 12-hour light period in our model (6).

To determine the rates of LPET and AET [V_{LPET} and V_{AET}, mol electron (mol C)$^{-1}$ s^{-1}], the fraction of photosynthetic electrons flowing into AET (f_{AET}, dimensionless) is introduced in our model, and f_{AET} is calculated in each time step to fulfill the intracellular immediate requirement of ATP and NADPH (7).

$$V_{LPET} = V_{PET} \cdot (1 - f_{AET}), \tag{S2}$$

$$V_{AET} = V_{PET} \cdot f_{AET}. \tag{S3}$$

The NADPH production rate [V_{NAPDH}, mol NADPH (mol C)$^{-1}$ s^{-1}] is:

$$V_{NAPDH} = V_{LPET} \cdot q_{LPET}^{NADPH}, \tag{S4}$$

where q_{LPET}^{NADPH} = 0.5 mol NADPH (mol electron)$^{-1}$ is the ratio of NADPH to electron in LPET (4).

The ATP production rate [V_{ATP}, mol ATP (mol C)$^{-1}$ s^{-1}] is:

$$V_{ATP} = V_{LPET} \cdot q_{LPET}^{ATP} + V_{AET} \cdot q_{AET}^{ATP}, \tag{S5}$$

where $q^{ATP}_{LPET} = q^{ATP}_{AET}$ = 0.65 mol ATP (mol electron)$^{-1}$ ratios of ATP to electron in LPET and AET (2-4).

The O_2 production rate [V_{O_2}, mol O_2 (mol C)$^{-1}$ s^{-1}] is:

$$V_{O_2} = V_{LPET} \cdot q^{O_2}_{LPET}, \tag{S6}$$

where $q^{O_2}_{LPET}$ = 0.25 mol O_2 (mol electron)$^{-1}$ is the ratio of O_2 to electron in LPET (3, 4).

2. Photorespiration

Photorespiration is a light-dependent process, which consumes ATP (adenosine triphosphate), NADPH (nicotinamide adenine dinucleotide phosphate hydrogen), O_2 and organic carbon, and produces CO_2 (8).

The maximal photorespiration rate [V^{max}_{PR}, mol C (mol C)$^{-1}$ s^{-1}] is computed based on the consumption that produced ATP by PET is fully consumed by photorespiration:

$$V^{max}_{PR} = \frac{V_{ATP}}{q^{ATP}_{PR}}, \tag{S7}$$

where q^{ATP}_{PR} = 7 mol ATP (mol C)$^{-1}$ is ATP to C ratio in photorespiration (8).

The rate of photorespiration [V_{PR}, mol C (mol C)$^{-1}$ s^{-1}] is also regulated by intracellular carbohydrate [CH_2O, mol C (mol C)$^{-1}$] and O_2 [O_2, mol O_2 m^{-3}]:

$$V_{PR} = V^{max}_{PR} \cdot \frac{CH_2O}{CH_2O + k^{PR}_{CH_2O}} \cdot \frac{O_2}{O_2 + k^{PR}_{O_2}}, \tag{S8}$$

where $k^{PR}_{CH_2O}$ = 0.4 [mol C (mol C)$^{-1}$] and $k^{PR}_{O_2}$ = 1.92 (mol O_2 m^{-3}) are half-saturating coefficients of CH_2O and O_2 for photorespiration (Fig. S1B).

The NADPH, ATP and O_2 consumption rates of photorespiration [V^{PR}_{NADPH}, V^{PR}_{ATP} and $V^{PR}_{O_2}$, mol NADPH (mol C)$^{-1}$ s^{-1}, mol ATP (mol C)$^{-1}$ s^{-1} and mol O_2 (mol C)$^{-1}$ s^{-1}] are:

$$V^{PR}_{NADPH} = V_{PR} \cdot q^{NADPH}_{PR}, \tag{S9}$$

$$V^{PR}_{ATP} = V_{PR} \cdot q^{ATP}_{PR}, \tag{S10}$$

$$V^{PR}_{O_2} = V_{PR} \cdot q^{O_2}_{C,PR}, \tag{S11}$$

where q^{NADPH}_{PR} = 4 mol NADPH (mol C)$^{-1}$ and $q^{O_2}_{C,PR}$ = 3 mol O_2 (mol C)$^{-1}$ are NADPH to C and O_2 to C ratios in photorespiration (8).

3. N_2 fixation

N_2 fixation requires both ATP and NADPH (9, 10). The maximal N_2 fixation rate [V_{NF}^{max}, mol N (mol C)$^{-1}$ s^{-1}] is calculated based on the assumption that produced ATP of PET are completely consumed by N_2 fixation.

$$V_{NF}^{max} = \frac{V_{ATP}}{q_{NF}^{ATP}}, \tag{S12}$$

where q_{NF}^{ATP} = 9 mol ATP (mol N)$^{-1}$ is ATP:N ratio in N_2 fixation (9, 10).

The rate [V_{NF}, mol N (mol C)$^{-1}$ s^{-1}] is also regulated by the Fe quota in nitrogenase [Fe_{NF}, μmol Fe (mol C)$^{-1}$] and inhibited by intracellular O_2.

$$V_{NF} = V_{NF}^{max} \cdot \frac{Fe_{NF}}{Fe_{NF} + k_{Fe}^{NF}} \cdot (1 - \frac{O_2}{O_2 + k_{O_2}^{NF}}), \tag{S13}$$

where k_{Fe}^{NF} [μmol Fe (mol C)$^{-1}$] and $k_{O_2}^{NF}$ (mol O_2 m^{-3}) are half-saturating coefficients of Fe_{NF} and O_2 for N_2 fixation.

The NADPH and ATP consumption rates of N_2 fixation [V_{NADPH}^{NF} and V_{ATP}^{NF}, mol NADPH (mol C)$^{-1}$ s^{-1} and mol ATP (mol C)$^{-1}$ s^{-1}] are:

$$V_{NADPH}^{NF} = V_{NF} \cdot q_{NF}^{NADPH}, \tag{S14}$$

$$V_{ATP}^{NF} = V_{NF} \cdot q_{NF}^{ATP}. \tag{S15}$$

where q_{NF}^{NADPH} = 3 mol NADPH (mol N)$^{-1}$ is NADPH:N ratio in N_2 fixation (9, 10).

4. CO_2 concentrating mechanism and carbon fixation

The energy requirement of the CO_2 concentrating mechanism (CCM) [q_{CCM}^{ATP} = 0.8 mol ATP (mol C)$^{-1}$] is calculated based on the fraction of leakage (50%) of inorganic carbon (C_i), the fraction of HCO_3^- in C_i (80%), and the cost for per HCO_3^- transportation [0.5 mol ATP (mol C)$^{-1}$] (11, 12).

Energy consumption rate of CCM [V_{ATP}^{CCM}, mol ATP (mol C)$^{-1}$ s^{-1}] is:

$$V_{ATP}^{CCM} = V_{CF} \cdot q_{CCM}^{ATP}, \tag{S16}$$

where V_{CF} [mol C (mol C)$^{-1}$ s^{-1}] is carbon fixation rate.

Carbon fixation also requires both NADPH and ATP (13), and consumption rates [V_{NADPH}^{CF} and V_{ATP}^{CF}, mol NADPH (mol C)$^{-1}$ s^{-1} and mol ATP (mol C)$^{-1}$ s^{-1}] are:

$$V_{NADPH}^{CF} = V_{CF} \cdot q_{CF}^{NADPH}, \tag{S17}$$

$$V_{ATP}^{CF} = V_{CF} \cdot q_{CF}^{ATP}. \tag{S18}$$

V_{CF} is solved at each time step with f_{AET}, based on the consumption that total NADPH and ATP production by PET are immediately and fully utilized by intracellular process:

$$V_{NADPH} = V_{NADPH}^{PR} + V_{NADPH}^{CF} + V_{NADPH}^{NF}, \quad \text{(S19)}$$

$$V_{ATP} = (V_{ATP}^{CCM} + V_{ATP}^{PR} + V_{ATP}^{CF} + V_{ATP}^{NF}) \cdot (1 + \gamma_{MT}), \quad \text{(S20)}$$

where γ_{MT} (dimensionless) represents the ratio of ATP consumption by maintenance to other processes.

The carbon skeleton production rate [V_{CS}, mol C (mol C)$^{-1}$ s^{-1}] is stimulated by carbohydrate and downregulated by its own accumulation [CS, mol C (mol C)$^{-1}$]:

$$V_{CS} = v_{CS}^{max} \cdot \frac{CH_2O}{CH_2O + k_{CH_2O}^{CS}} \cdot \frac{CS_{max} - CS}{CS_{max}}, \quad \text{(S21)}$$

where v_{CS}^{max} [mol C (mol C)$^{-1}$ s^{-1}] is the maximal production rate of the carbon skeleton, $k_{CH_2O}^{CS}$ [mol C (mol C)$^{-1}$] is the half-saturation constant of carbohydrates for carbon skeleton production, and CS_{max} [mol C (mol C)$^{-1}$] is the maximum CS storage.

5. Respiratory protection

Respiratory protection (RP) rate is regulated by the demand for N_2 fixation and intracellular O_2 (7, 14, 15):

$$V_{RP} = v_{RP}^{max} \cdot (1 - e^{-\alpha_I \cdot I}) \cdot \frac{CS}{CS + k_{CS}} \cdot \left(\frac{N_{max} - N}{N_{max}}\right) \cdot \frac{O_2}{O_2 + k_{O_2}^{NF}}, \quad \text{(S22)}$$

where v_{RP}^{max} [mol C (mol C)$^{-1}$ s^{-1}] is the maximal respiratory protection rate, k_{CS} [mol C (mol C)$^{-1}$] is the half-saturating coefficient of the carbon skeleton for respiratory protection, and N_{max} [mol N (mol C)$^{-1}$] is the maximal N storage.

The O_2 consumption by RP [$V_{O_2}^{RP}$, mol O_2 (mol C)$^{-1}$ s^{-1}] is:

$$V_{O_2}^{RP} = V_{RP} \cdot q_C^{O_2}, \quad \text{(S23)}$$

where $q_C^{O_2}$ [mol O_2 (mol C)$^{-1}$] is the ratio of O_2 to carbon in carbohydrate respiration.

6. O_2 diffusion

The ratio (ε, dimensionless) of the O_2 diffusion coefficient of the cell membrane relative to that in seawater (d_{O_2}, m^2 s^{-1}) (i.e., parameter measuring the O_2 permeability of the membrane) is considered to change throughout the daytime (16). ε is assumed to increase with the increase of intracellular O_2 concentration:

$$\varepsilon = \varepsilon_{max} \cdot \frac{O_2}{O_2 + k_{O_2}^{diff}}, \quad \text{(S24)}$$

where ε_{max} is the maximal relative diffusion coefficient and $k_{O_2}^{diff}$ (mol O_2 m^{-3}) is the half-saturation constant of O_2 for ε.

The rate of O_2 diffusion (T_{O_2}, mol O_2 m^{-3} s^{-1}) between intracellular cytoplasm and ambient environment is parameterized by adopting the scheme of (17):

$$T_{O_2} = \frac{-2\cdot\pi\cdot d_{O_2}\cdot L}{V} \cdot \left\{\frac{1}{\varepsilon}\cdot\ln\left(\frac{R}{R+L_g}\right) - \ln\left(\frac{R+L_g+L_b}{R+L_g}\right)\right\}^{-1} \cdot (O_2^E - O_2), \qquad \text{(S25)}$$

where L (m) and V (m^3) are the length and the volume of the trichome, R (m) is the radius of the cytoplasm, L_g (m) is the thickness of the cell membrane, L_b (m) is the thickness of the boundary layer, O_2^E is the ambient far-field O_2 concentration.

7. Intracellular Fe pools and translocation

Trichodesmium can uptake more Fe than that required by its metabolism (called 'luxury uptake') especially in high-Fe environments, and the excess Fe is stored for surviving in low Fe environments (18, 19). Therefore, the total intracellular Fe quota [Fe, μmol Fe (mol C)$^{-1}$] consists of Fe in metabolism and storage [Fe_M and Fe_{ST}, μmol Fe (mol C)$^{-1}$], calculated based on the threshold of Fe [Fe_{TH}, μmol Fe (mol C)$^{-1}$] (20).

$$Fe_M = Fe, \qquad \text{when } Fe \le Fe_{TH}, \qquad \text{(S26)}$$

$$Fe_M = Fe_{TH} + (1 - f_{ST}) \cdot (Fe - Fe_{TH}), \text{ when } Fe > Fe_{TH}, \qquad \text{(S27)}$$

where f_{ST} (dimensionless) is the fraction of luxury Fe uptake.

Fe allocations are among Fe_M, including Fe in photosystems, active nitrogenase, inactivated nitrogenase, maintenance and buffer [Fe_{PS}, Fe_{NF}, Fe_{NF}^{NA}, Fe_{MT} and Fe_{BF}, μmol Fe (mol C)$^{-1}$] (Fig. 1B). Fe in maintenance is set diurnally constant at 10% of Fe_M (20). Fe used in the photosystems and nitrogenase is from the buffer pool (15).

The synthesis rate of photosystems [T_{PS}^{BF}, μmol Fe (mol C)$^{-1}$ s^{-1}] is stimulated by light intensity and is gradually saturated with Fe_{PS}:

$$T_{PS}^{BF} = TF_{PS_{max}}^{BF} \cdot (1 - e^{-\alpha_I \cdot I}) \cdot \left(1 - \frac{Fe_{PS}}{Fe_{PS} + k_{Fe_{PS}}^{PS_{syn}}}\right), \qquad \text{(S28)}$$

where $T_{PS_{max}}^{BF}$ [μmol Fe (mol C)$^{-1}$ s^{-1}] is the maximal synthesis rate of photosystems, $k_{Fe_{PS}}^{PS_{syn}}$ [μmol Fe (mol C)$^{-1}$] is the half-saturating coefficients of Fe_{PS} for the synthesis of photosystems.

The decomposition rate of photosystems [T_{BF}^{PS}, μmol Fe (mol C)$^{-1}$ s^{-1}] is stimulated by Fe_{PS} but inhibited by respiratory protection (5):

$$T_{BF}^{PS} = T_{BF_{max}}^{PS} \cdot \frac{Fe_{PS}}{Fe_{PS} + k_{Fe_{PS}}^{PS_{dec}}} \cdot e^{-\beta \cdot V_{RP}}, \tag{S29}$$

where $T_{BF_{max}}^{PS}$ [μmol Fe (mol C)$^{-1}$ s^{-1}] is the maximal decomposition rate of photosystems, $k_{Fe_{PS}}^{PS_{dec}}$ [μmol Fe (mol C)$^{-1}$] is the half-saturating coefficient of Fe_{PS} for the decomposition of photosystems. Fe released from decomposed photosystems returns to buffer pool (15).

For nitrogenase, intracellular requirement for N_2 fixation and Fe_{BF} regulate its synthesis rate [T_{NF}^{BF}, μmol Fe (mol C)$^{-1}$ s^{-1}]:

$$T_{NF}^{BF} = T_{NF_{max}}^{BF} \cdot (1 - e^{-\alpha_I \cdot I}) \cdot \frac{CS}{CS + k_{CS}} \cdot (\frac{N_{max} - N}{N_{max}}) \cdot \frac{Fe_{BF}}{Fe_{BF} + k_{Fe_{BF}}^{NF_{syn}}}, \tag{S30}$$

where $T_{NF_{max}}^{BF}$ [μmol Fe (mol C)$^{-1}$ s^{-1}] is the maximal nitrogenase synthesis rate, k_{CS} [mol C (mol C)$^{-1}$] and $k_{Fe_{BF}}^{NF_{syn}}$ [μmol Fe (mol C)$^{-1}$] are half-saturating coefficients of the carbon skeleton and Fe_{BF} for the synthesis of nitrogenase, respectively.

The decomposition of nitrogenase seems to occur at night (21, 22), and therefore it is not considered during the light period in our model. Notably, nitrogenase is inhibited upon exposure to O_2, flowing into the pool of inactivated nitrogenase (5) at the rate [T_{NF}^{NA}, μmol Fe (mol C)$^{-1}$ s^{-1}]:

$$T_{NF}^{NA} = T_{NF_{max}}^{NA} \cdot \frac{Fe_{NF}}{Fe_{NF} + k_{Fe}^{NF}} \cdot \frac{O_2}{O_2 + k_{O_2}^{NF}}, \tag{S31}$$

where $T_{NF_{max}}^{NA}$ [μmol Fe (mol C)$^{-1}$ s^{-1}] is the maximal inactivation rate of nitrogenase.

The Fe in nitrogenase and photosystems from (21) were estimated from observed protein content, based on Fe atoms in per protein. PSII, Cyt *b6f*, PSI and Ferredoxin together represents photosystems. Cyt *b6f* and Ferredoxin were not measured in (21) but estimated by assuming Cyt *b6f*:PSII = 1:1 in Fe quota and Ferredoxin:PSI = 1:1 in protein content. Further details are in the supplementary information in (20).

8. Integration of state variables during the daytime

The diurnal change rates, which are normalized to carbon biomass, of CH_2O, CS, N, intracellular O_2 and Fe are represented in ordinary differential equations (ODEs). Note that O_2 is in a unit volumetric concentration (mol O_2 m^{-3}):

$$\frac{dCH_2O}{dt} = V_{CF} - V_{CS} - V_{PR} - V_{RP}, \tag{S32}$$

$$\frac{dCS}{dt} = V_{CS}, \tag{S33}$$

$$\frac{dN}{dt} = V_{NF}, \quad (S34)$$

$$\frac{dO_2}{dt} = (V_{O_2} - V^{PR}_{O_2} - V^{RP}_{O_2}) \cdot Q_C + T_{O_2}, \quad (S35)$$

$$\frac{dFe_{PS}}{dt} = T^{BF}_{PS} - T^{PS}_{BF}, \quad (S36)$$

$$\frac{dFe_{NF}}{dt} = T^{BF}_{NF} - T^{NA}_{NF}, \quad (S37)$$

$$\frac{dFe^{NA}_{NF}}{dt} = T^{NA}_{NF}, \quad (S38)$$

$$\frac{dFe_{BF}}{dt} = T^{PSI}_{BF} - T^{BF}_{PSI} \cdot + T^{BF}_{NF}, \quad (S39)$$

where Q_C = 18333 mol C m^{-3} is the cellular carbon biomass quota (23). ODEs are run over a12-hour light period with ode15s integrator of MATLAB (24).

9. Biosynthesis and growth rate

Trichodesmium might store newly fixed C and N during the daytime and assimilate them into biomass, mainly during the dark period (25). Therefore, for simplicity, no biomass is synthesized during the light period in the model. Instead, the model calculates the amount of biomass [*Bio*, mol C (mol C)$^{-1}$] that can be synthesized using the carbohydrates, carbon skeletons and fixed N at the end of the light period. *Bio* is the smaller of N-based (Bio_N) and C-based biomass (Bio_C), with Bio_N calculated by dividing fixed N to the molar N:C (0.159) (26). Bio_C is calculated from the carbohydrates and carbon skeleton considering mass and energy balance. The energy needed for biosynthesis is from the respiration of carbohydrates ($CH_2O^{RESP}_{BIO}$):

$$Bio_C \cdot q^{ATP}_{BIO} \cdot (1 + \gamma_{MT}) = CH_2O^{RESP}_{BIO} \cdot q^{ATP}_{RESP}, \quad (S40)$$

where q^{ATP}_{BIO} = 2 mol ATP (mol C)$^{-1}$ is the ATP requirement rate by biosynthesis (14), and q^{ATP}_{RESP} = 5 mol ATP (mol C)$^{-1}$ is the ATP production rate from respiring carbohydrates (27). Meanwhile, the non-respired carbohydrates and all the carbon skeletons are involved in biosynthesis:

$$Bio_C = CH_2O - CH_2O^{RESP}_{BIO} + CS. \quad (S41)$$

Bio_C then can be solved from the above two equations. Note that the carbohydrate respiration calculated in this step is counted in the daily integrated respiration as the ordinary respiration.

Noting that all the rates have been normalized to carbon biomass, *Bio* is therefore the relative increase in biomass over one day. The growth rate (*G*) is then the natural log of (1 + *Bio*) divided by 1 day.

SUPPLEMENTARY REFERENCES

1. Milligan AJ, Berman-Frank I, Gerchman Y, Dismukes GC, Falkowski PG. 2007. Light-dependent oxygen consumption in nitrogen-fixing cyanobacteria plays a key role in nitrogenase protection. Journal of Phycology 43:845-852.
2. Behrenfeld MJ, Milligan AJ. 2013. Photophysiological expressions of iron stress in phytoplankton. Annual Review of Marine Science 5:217-246.
3. Geider RJ, Moore CM, Ross ON. 2009. The role of cost–benefit analysis in models of phytoplankton growth and acclimation. Plant Ecology and Diversity 2:165-178.
4. Allen JF. 2003. Cyclic, pseudocyclic and noncyclic photophosphorylation: New links in the chain. Trends in Plant Science 8:15-19.
5. Berman-Frank I, Lundgren P, Chen YB, Kupper H, Kolber Z, Bergman B, Falkowski P. 2001. Segregation of nitrogen fixation and oxygenic photosynthesis in the marine cyanobacterium *Trichodesmium*. Science 294:1534-1537.
6. Reimers AM, Knoop H, Bockmayr A, Steuer R. 2017. Cellular trade-offs and optimal resource allocation during cyanobacterial diurnal growth. Proceedings of the National Academy of Sciences of the United States of America 114:E6457-E6465.
7. Luo W, Inomura K, Zhang H, Luo Y-W. 2022. N_2 fixation in *Trichodesmium* does not require spatial segregation from photosynthesis. mSystems 7:e00538-22.
8. Bauwe H, Hagemann M, Fernie AR. 2010. Photorespiration: players, partners and origin. Trends in Plant Science 15:330-336.
9. Flores E, Herrero A. 1994. Assimilatory nitrogen metabolism and its regulation, p 487-517. *In* Bryant DA (ed), The Molecular Biology of Cyanobacteria. Kluwer Academic Publishers, Dordrecht.
10. Flores E, Frías JE, Rubio LM, Herrero A. 2005. Photosynthetic nitrate assimilation in cyanobacteria. Photosynthesis Research 83:117-133.
11. Eichner M, Thoms S, Kranz SA, Rost B. 2015. Cellular inorganic carbon fluxes in *Trichodesmium*: A combined approach using measurements and modelling. Journal of Experimental Botany 66:749-759.
12. Raven JA, Beardall J, Giordano M. 2014. Energy costs of carbon dioxide concentrating mechanisms in aquatic organisms. Photosynthesis Research 121:111-124.
13. Baker NR, Harbinson J, Kramer DM. 2007. Determining the limitations and regulation of photosynthetic energy transduction in leaves. Plant Cell and Environment 30:1107-1125.
14. Inomura K, Wilson ST, Deutsch C. 2019. Mechanistic model for the coexistence of nitrogen fixation and photosynthesis in marine *Trichodesmium*. mSystems 4:e00210-19.
15. Inomura K, Deutsch C, Wilson ST, Masuda T, Lawrenz E, Lenka B, Sobotka R, Gauglitz JM, Saito MA, Prášil O, Follows MJ. 2019. Quantifying oxygen management and temperature and light dependencies of nitrogen fixation by *Crocosphaera watsonii*. mSphere 4:e00531-19.
16. Cornejo-Castillo FM, Zehr JP. 2019. Hopanoid lipids may facilitate aerobic nitrogen fixation in the ocean. Proceedings of the National Academy of Sciences of the United States of America 116:18269-18271.
17. Staal M, Meysman FJ, Stal LJ. 2003. Temperature excludes N_2-fixing heterocystous cyanobacteria in the tropical oceans. Nature 425:504-507.
18. Kustka AB, Sañudo-Wilhelmy SA, Carpenter EJ, Capone D, Burns J, Sunda WG. 2003. Iron requirements for dinitrogen- and ammonium-supported growth in cultures of *Trichodesmium* (IMS 101): Comparison with nitrogen fixation rates and iron: carbon ratios of field populations. Limnology and Oceanography 48:1869–1884.
19. Berman-Frank I, Cullen JT, Shaked Y, Sherrell RM, Falkowski PG. 2001. Iron availability, cellular iron quotas, and nitrogen fixation in *Trichodesmium*. Limnology and Oceanography 46:1249-1260.

20. Luo YW, Shi D, Kranz SA, Hopkinson BM, Hong H, Shen R, Zhang F. 2019. Reduced nitrogenase efficiency dominates response of the globally important nitrogen fixer *Trichodesmium* to ocean acidification. Nature Communications 10:1521.
21. Shi D, Kranz SA, Kim JM, Morel FM. 2012. Ocean acidification slows nitrogen fixation and growth in the dominant diazotroph *Trichodesmium* under low-iron conditions. Proceedings of the National Academy of Sciences of the United States of America 109:E3094-100.
22. Capone DG, O'Neil JM, Zehr JP, Carpenter EJ. 1990. Basis for diel variation in nitrogenase activity in the marine planktonic cyanobacterium *Trichodesmium thiebautii*. Applied and Environmental Microbiology 56:3532-3536.
23. Bratbak G, Dundas I. 1984. Bacterial dry matter content and biomass estimations. Applied and Environmental Microbiology 48:755-757.
24. Shampine LF, Reichelt MW. 1997. The MATLAB ODE Suite. SIAM J Sci Comput 18:1-22.
25. Finzi-Hart JA, Pett-Ridge J, Weber PK, Popa R, Fallon SJ, Gunderson T, Hutcheon ID, Nealson KH, Capone DG. 2009. Fixation and fate of C and N in the cyanobacterium *Trichodesmium* using nanometer-scale secondary ion mass spectrometry. Proceedings of the National Academy of Sciences of the United States of America 106:6345-6350.
26. LaRoche J, Breitbarth E. 2005. Importance of the diazotrophs as a source of new nitrogen in the ocean. Journal of Sea Research 53:67-91.
27. Mitchell P. 1970. Aspects of the chemiosmotic hypothesis. Biochemical Journal 116:5-6.
28. Luo W, Luo Y-W. 2023. Diurnally dynamic iron allocation promotes N_2 fixation in marine dominant diazotroph *Trichodesmium*. Computational and Structural Biotechnology Journal 21:3503-3512.
29. Benson BB, Krause D. 1984. The concentration and isotopic fractionation of oxygen dissolved in freshwater and seawater in equilibrium with the atmosphere. Limnology and Oceanography 29:620-632.
30. Carpenter EJ, Oneil JM, Dawson R, Capone DG, Siddiqui PJA, Roenneberg T, Bergman B. 1993. The tropical diazotrophic phytoplankter *Trichodesmium*: Biological characteristics of two common species. Marine Ecology Progress Series 95:295-304.

TABLE S1 Optimized parameters

Symbol	Unit	Definition	Value			
			Low Fe (40 pM)		High Fe (1250 pM)	
			Fixed permeability	Dynamic permeability	Fixed permeability	Dynamic permeability
v_{RP}^{max}	mol C (mol C)$^{-1}$ s^{-1}	Maximal respiratory protection rate	9.6×10^{-4}	1.2×10^{-3}	9.8×10^{-4}	8.2×10^{-4}
$T_{PS_{max}}^{BF}$	μmol Fe (mol C)$^{-1}$ s^{-1}	Maximal synthesis rate of photosystems	5.7×10^{-6}	3.8×10^{-6}	2.0×10^{-6}	1.6×10^{-5}
$T_{BF_{max}}^{PS}$	μmol Fe (mol C)$^{-1}$ s^{-1}	Maximal decomposition rate of photosystems	6.3×10^{-4}	1.3×10^{-3}	1.7×10^{-3}	4.8×10^{-3}
$T_{NF_{max}}^{BF}$	μmol Fe (mol C)$^{-1}$ s^{-1}	Maximal synthesis rate of nitrogenase	2.2×10^{-2}	2.8×10^{-2}	2.7×10^{-2}	2.8×10^{-2}

TABLE S2 Fixed model parameters

Symbol	Unit	Definition	Value	Source or note
v_{PET}^{max}	mol electron (mol C)$^{-1}$ s^{-1}	Maximal PET rate	1.0×10^{-2}	This study [a]
ε_{max}	dimensionless	Maximal relative diffusivity of cell membrane	2×10^{-4}	This study [a]
$k_{O_2}^{diff}$	mol O_2 m^{-3}	Half-saturating coefficient of O_2 for relative diffusivity of cell membrane	0.213	This study [a]
$k_{O_2}^{PR}$	mol O_2 m^{-3}	Half-saturating coefficient of O_2 for photorespiration	1.917	This study [a]
$k_{CH_2O}^{PR}$	mol C (mol C)$^{-1}$	Half-saturating coefficient of CH_2O for photorespiration	0.4	This study [a]
v_{CS}^{max}	mol C (mol C)$^{-1}$ s^{-1}	Maximal production rate of CS	8.6×10^{-6}	(28)
k_{CS}	mol C (mol C)$^{-1}$	Half-saturating coefficient of CS for RP	0.4	(28)
$k_{CH_2O}^{CS}$	mol C (mol C)$^{-1}$	Half-saturating coefficient of CH_2O for CS production	0.4	(28)
N_{max}	mol N (mol C)$^{-1}$	Maximal fixed storage	0.159 [b]	(28)
CS_{max}	mol C (mol C)$^{-1}$	Maximal CS storage	1 [c]	(28)
α_I	μmol^{-1} m^2 s	Initial slope of P versus I curve	0.01	(14)
β	(mol C)$^{-1}$ mol C s	Parameter of inhibition effect of respiration on PET	2×10^{4}	(7)
$k_{O_2}^{NF}$	mol O_2 m^{-3}	Half-saturating coefficient of O_2 for N_2 fixation	0.01	(7)
d_{O_2}	m^2 s^{-1}	O_2 diffusion coefficient at 34 PSU and 25 °C	2.26×10^{-9}	(29)
$k_{Fe_{PS}}^{PS_{syn}}$	μmol Fe (mol C)$^{-1}$	Half-saturating coefficient of Fe_{PS} for the synthesis of photosystems	1.0	(28)
$k_{Fe_{PS}}^{PS_{dec}}$	μmol Fe (mol C)$^{-1}$	Half-saturating coefficient of Fe_{PS} for the decomposition of photosystems	25	(28)
$k_{Fe_{BF}}^{NF_{syn}}$	μmol Fe (mol C)$^{-1}$	Half-saturating coefficient of Fe_{BF} for the synthesis of nitrogenase	5.0	(28)
$T_{NF_{max}}^{NA}$	μmol Fe (mol C)$^{-1}$ s^{-1}	Maximal inactivation rate of nitrogenase	3.3×10^{-3}	(28)
k_{Fe}^{PS}	μmol Fe (mol C)$^{-1}$	Half-saturating coefficient of Fe in photosystems for PET rate	25	(28)
k_{Fe}^{NF}	μmol Fe (mol C)$^{-1}$	Half-saturating coefficient of Fe in nitrogenase for N_2 fixation	91	(20)
Fe_{TH}	μmol Fe (mol C)$^{-1}$	Threshold of intracellular metabolic Fe quota	24.4	(20)
f_{ST}	dimensionless	Fraction of luxury Fe uptake	0.90	(20)
γ_{MT}	dimensionless	Ratio of the energy consumed by maintenance to other process	10%	(20)
		Boundary conditions		
I_{max}	μmol m^{-2} s^{-1}	Maximal light intensity in fixed-Fe and dynamic-Fe cases under diurnally changing light intensity	160	
O_2^E	mol O_2 m^{-3}	Extracellular far-field O_2	0.213	
		Elemental or energy stoichiometries of metabolic activities		
q_{LPET}^{NADPH}	mol NADPH (mol electron)$^{-1}$	NADPH/electron ratio of LPET	0.5	(4)
q_{LPET}^{ATP}	mol ATP (mol electron)$^{-1}$	ATP/electron ratio of LPET	0.65	(3)
$q_{LPET}^{O_2}$	mol O_2 (mol electron)$^{-1}$	O_2/electron ratio of LPET	0.25	(4)
q_{AET}^{ATP}	mol ATP (mol electron)$^{-1}$	ATP/electron ratio of MR-AET	0.65	(3)
q_{NF}^{NADPH}	mol NADPH (mol N)$^{-1}$	NADPH/N ratio of N_2 fixation	3	(9, 10)

q_{NF}^{ATP}	mol ATP (mol N)$^{-1}$	ATP/N ratio of N_2 fixation	9	(9, 10)
q_{PR}^{NADPH}	mol NADPH (mol C)$^{-1}$	NADPH/C ratio of photorespiration	4	(8)
q_{PR}^{ATP}	mol ATP (mol C)$^{-1}$	ATP/C ratio of photorespiration	7	(8)
$q_{C,\,PR}^{O_2}$	mol O_2 (mol C)$^{-1}$	O_2/C ratio of photorespiration	3	(8)
q_{CF}^{NADPH}	mol NADPH (mol C)$^{-1}$	NADPH/C ratio of C fixation	2	(13)
q_{CF}^{ATP}	mol ATP (mol C)$^{-1}$	ATP/C ratio of C fixation	3	(13)
q_{CCM}^{ATP}	mol ATP (mol C)$^{-1}$	ATP/C ratio of CCM	0.8	(12)
q_{BIO}^{ATP}	mol ATP (mol C)$^{-1}$	ATP/C ratio of biosynthesis	2	(14)
q_{RESP}^{ATP}	mol ATP (mol C)$^{-1}$	ATP/C ratio of respiration	5	(27)
$q_{C}^{O_2}$	mol O_2 (mol C)$^{-1}$	O_2/C ratio of respiration	1	(27)
Q_C	mol C m^{-3}	Cellular carbon biomass concentration	18333	(23)
		Morphological parameters of Trichodesmium		
L	m	Length of the total trichome	554×10^{-6}	(30)
R	m	Radius of the cytoplasm	4.80×10^{-6}	(30)
L_g	m	Thickness of cell membrane layer	0.076	(30)

[a] Estimated based on model experiments under constant light intensity.

[b] By multiplying the initial C biomass with the molar N:C (0.159) of *Trichodesmium* (26).

[c] CS_{max} is set to be the same as the initial C biomass.

TABLE S3 Intermediate process and state variables

Symbol	Unit	Definition
		Intermediate process variables
V_{PET}	mol electron (mol C)$^{-1}$ s^{-1}	PET rate
V_{LPET}	mol electron (mol C)$^{-1}$ s^{-1}	LPET rate
V_{AET}	mol electron (mol C)$^{-1}$ s^{-1}	AET rate
f_{AET}	dimensionless	Fraction of electrons in PET to AET
V_{NAPDH}	mol NADPH (mol C)$^{-1}$ s^{-1}	NADPH production rate
V_{ATP}	mol ATP (mol C)$^{-1}$ s^{-1}	ATP production rate
V_{O_2}	mol O_2 (mol C)$^{-1}$ s^{-1}	O_2 production rate
V_{PR}	mol C (mol C)$^{-1}$ s^{-1}	Photorespiration rate
V_{NADPH}^{PR}	mol NADPH (mol C)$^{-1}$ s^{-1}	NADPH consumption rate of photorespiration
V_{ATP}^{PR}	mol ATP (mol C)$^{-1}$ s^{-1}	ATP consumption rate of photorespiration
$V_{O_2}^{PR}$	mol O_2 (mol C)$^{-1}$ s^{-1}	O_2 consumption rate of photorespiration
V_{NF}^{max}	mol N (mol C)$^{-1}$ s^{-1}	Maximal N_2 fixation rate
V_{NF}	mol N (mol C)$^{-1}$ s^{-1}	N_2 fixation rate
V_{NADPH}^{NF}	mol NADPH (mol C)$^{-1}$ s^{-1}	NADPH consumption rate of N_2 fixation
V_{ATP}^{NF}	mol ATP (mol C)$^{-1}$ s^{-1}	ATP consumption rate of N_2 fixation
V_{ATP}^{CCM}	mol ATP (mol C)$^{-1}$ s^{-1}	ATP consumption rate of CCM
V_{NADPH}^{CF}	mol NADPH (mol C)$^{-1}$ s^{-1}	NADPH consumption rate of C fixation
V_{ATP}^{CF}	mol ATP (mol C)$^{-1}$ s^{-1}	ATP consumption rate of C fixation
V_{CS}	mol C (mol C)$^{-1}$ s^{-1}	Carbon skeleton production rate
V_{RP}	mol C (mol C)$^{-1}$ s^{-1}	Respiratory protection rate
$V_{O_2}^{RP}$	mol O_2 (mol C)$^{-1}$ s^{-1}	O_2 consumption rates by respiratory protection
ε	dimensionless	Relative diffusivity of cell membrane
T_{O_2}	mol O_2 m^{-3} s^{-1}	O_2 diffusion rate between cytoplasm and ambient environment
Fe_M	µmol Fe (mol C)$^{-1}$	Intracellular Fe quota in metabolism
Fe_{ST}	µmol Fe (mol C)$^{-1}$	Intracellular Fe quota in storage
T_{PS}^{BF}	µmol Fe (mol C)$^{-1}$ s^{-1}	Synthesis rate of photosystems
T_{BF}^{PS}	µmol Fe (mol C)$^{-1}$ s^{-1}	Decomposition rate of photosystems
T_{NF}^{BF}	µmol Fe (mol C)$^{-1}$ s^{-1}	Synthesis rate of nitrogenase
T_{NF}^{NA}	µmol Fe (mol C)$^{-1}$ s^{-1}	Inactivation rate of nitrogenase
Bio	mol C (mol C)$^{-1}$	New synthesized biomass
Bio_N	mol C (mol C)$^{-1}$	New synthesized N-based biomass
Bio_C	mol C (mol C)$^{-1}$	New synthesized C-based biomass
$CH_2O_{BIO}^{RESP}$	mol C (mol C)$^{-1}$	Respired carbohydrates to fulfill the energy need for biosynthesis
G	d^{-1}	Specific growth rate
		State variables
CH_2O	mol C (mol C)$^{-1}$	Carbohydrate
CS	mol C (mol C)$^{-1}$	Carbon skeleton
N	mol N (mol C)$^{-1}$	Fixed N
O_2	mol O_2 m^{-3}	Intracellular O_2
Fe_{PS}	µmol Fe (mol C)$^{-1}$	Fe in photosystems
Fe_{NF}	µmol Fe (mol C)$^{-1}$	Fe in active nitrogenase
Fe_{NF}^{NA}	µmol Fe (mol C)$^{-1}$	Fe in inactivated nitrogenase
Fe_{BF}	µmol Fe (mol C)$^{-1}$	Fe in buffer

Note: The initial values (t = 0) of CH_2O, CS and N are set to be 0, and initial O_2 concentration is the same as that of ambient O_2 (0.213 mol O_2 m^{-3}).

TABLE S4 Comparison of modeled and observed growth rates under diurnally constant light intensity

Growth rate (d^{-1})	Low Fe (40 pM)	High Fe (1250 pM)
Observation*	0.26 ± 0.02	0.46 ± 0.01
Model	0.28	0.45

* Data are from (21).

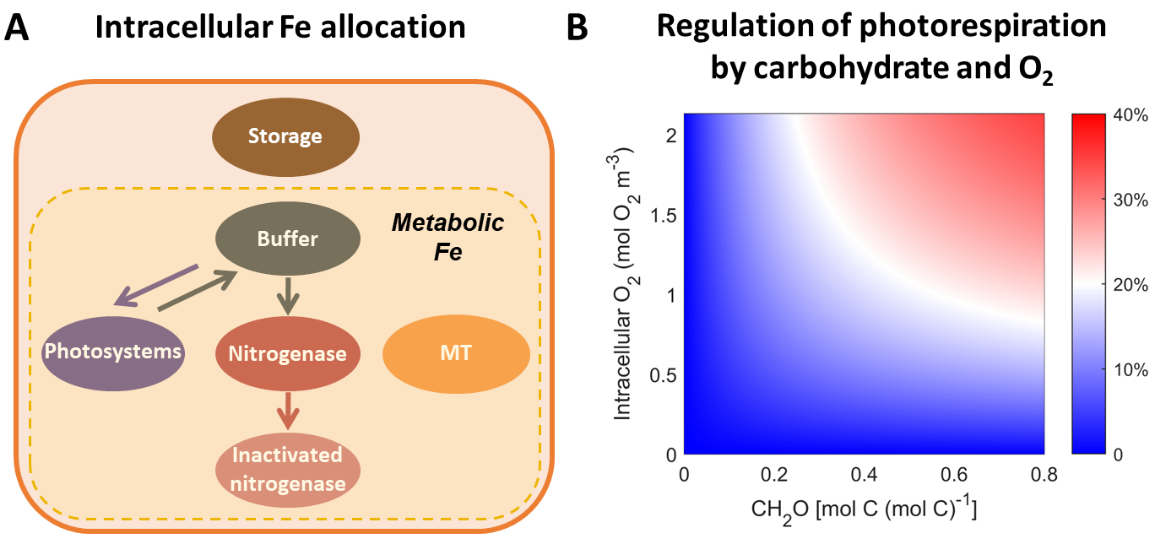

FIG S1 (A) Model structure of intracellular Fe allocation in *Trichodesmium* and (B) regulation of photorespiration by carbohydrate and O_2. (**A**) The model encompasses fundamental intracellular Fe pools, including storage and five metabolic pools (photosystems, active and inactivated nitrogenase, maintenance, buffer and storage). Note that all inactivated nitrogenase by O_2 does not decompose and Fe in it is not recycled during model period (daytime). (**B**) The regulation effect is represented by the ratio of V_{PR} to V_{PR}^{max}. The values $k_{CH_2O}^{PR}$ = 0.4 [mol C (mol C)$^{-1}$] and $k_{O_2}^{PR}$ = 1.92 (mol O_2 m^{-3}) were determined through model optimization to best reconcile simulated and observed daily patterns of photosynthesis, nitrogenase, and growth rates in *Trichodesmium* (21).

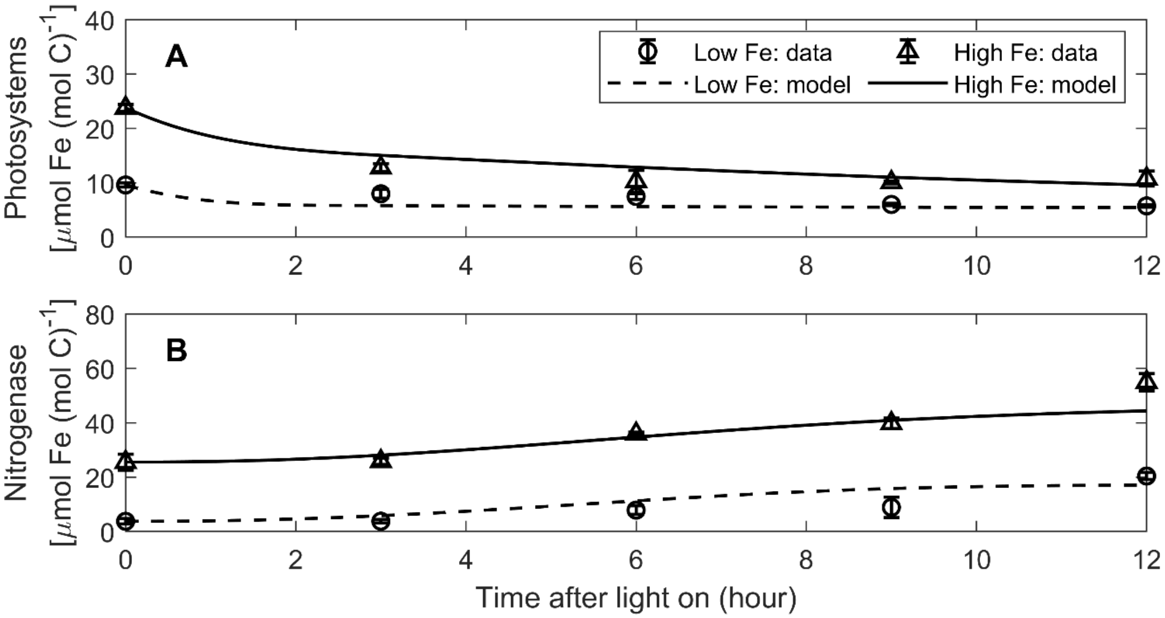

FIG S2 Modeled and observed diurnal variations of Fe in photosystems and nitrogenase. The observational data are from (21). The model simulations were performed with dynamic O_2 permeability of the cell membrane under both low-Fe and high-Fe conditions. Light intensity in the model is diurnally constant as that in (21). Error bars represent one standard deviation of observations (21).

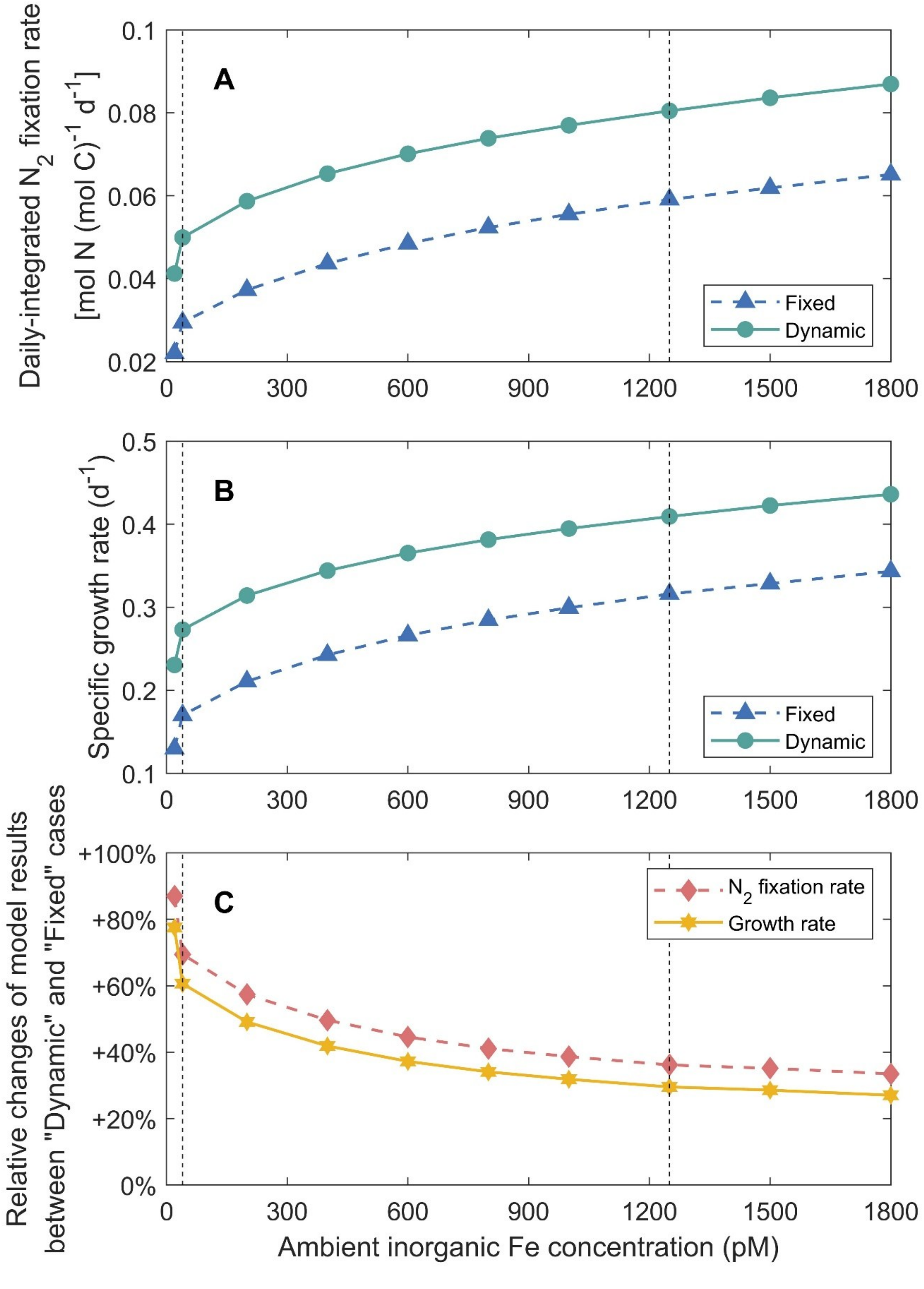

FIG S3 Simulated daily-integrated N_2 fixation rates (A), growth rates (B) and their relative changes (C) between "Dynamic permeability" and "Fixed permeability" cases under different ambient inorganic iron concentrations. Black dashed lines represent ambient inorganic iron concentrations (40 and 1250 pM) used in the standard model cases.

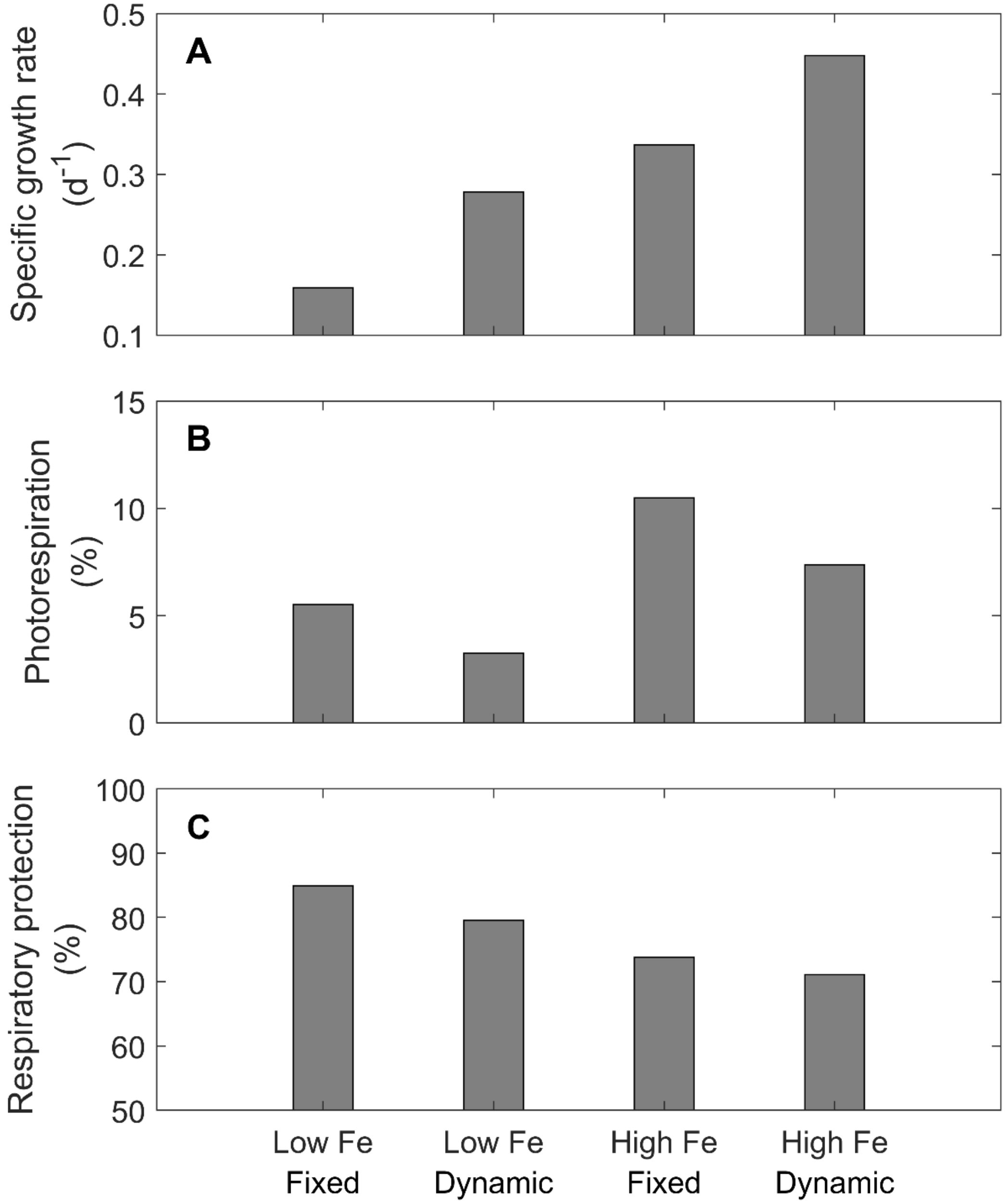

FIG S4 Results of model experiments under the constant light intensity, including growth rates (A), gross fixed carbon allocated to respiratory protection (B) and photorespiration (C). The model simulations were performed with diurnally fixed or dynamic O_2 permeability of the cell membrane under low-Fe (40 pM) and high-Fe (1250 pM) conditions.

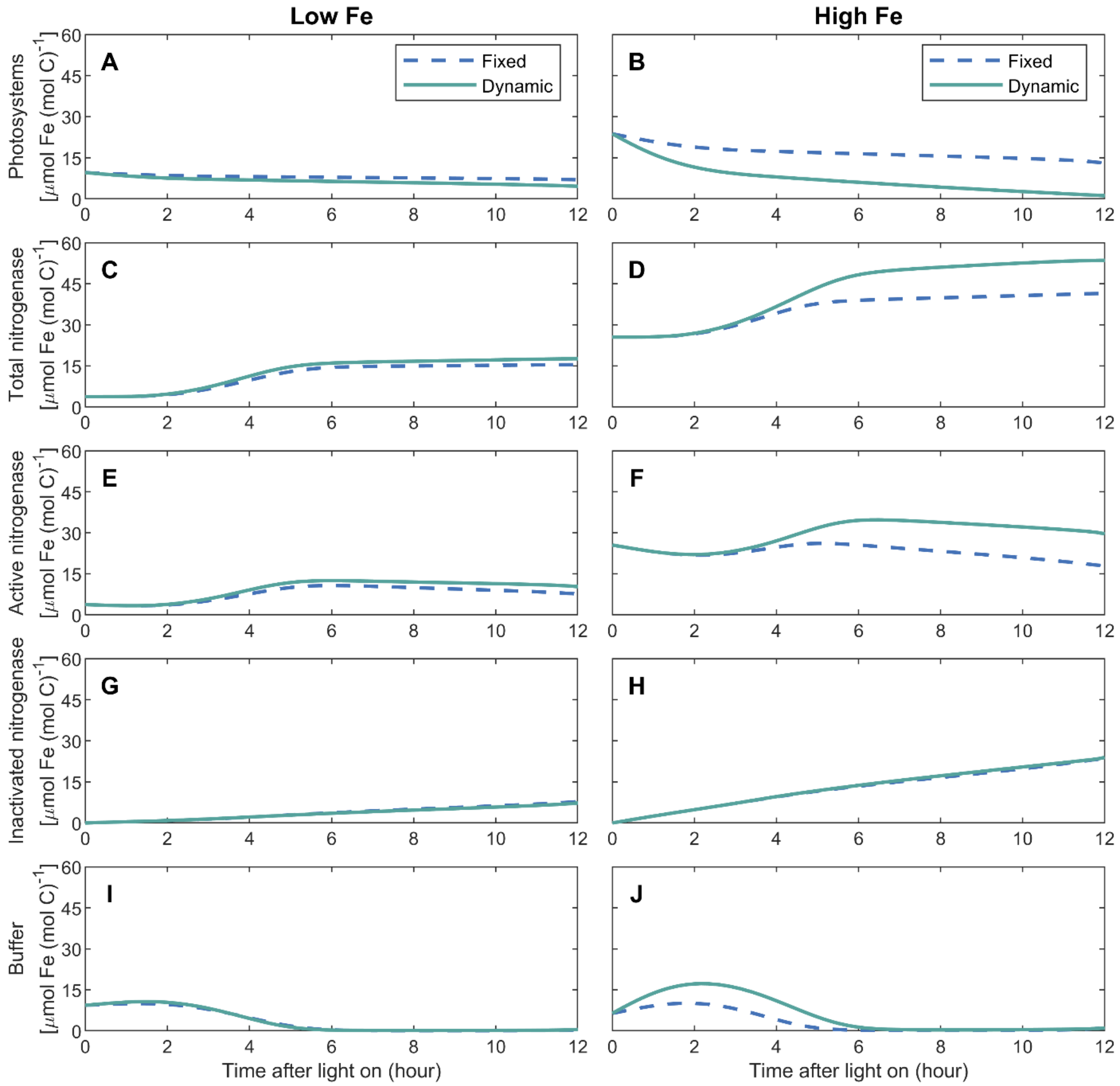

FIG S5 Simulated diurnal variations of Fe in photosystems, total, active and inactivated nitrogenase, and buffer. The model is simulated with diurnally fixed or dynamic O_2 permeability of the cell membrane under low-Fe (40 pM) (**A**, **C**, **E**, **G** and **I**) and high-Fe (1250 pM) (**B**, **D**, **F**, **H** and **J**) conditions.

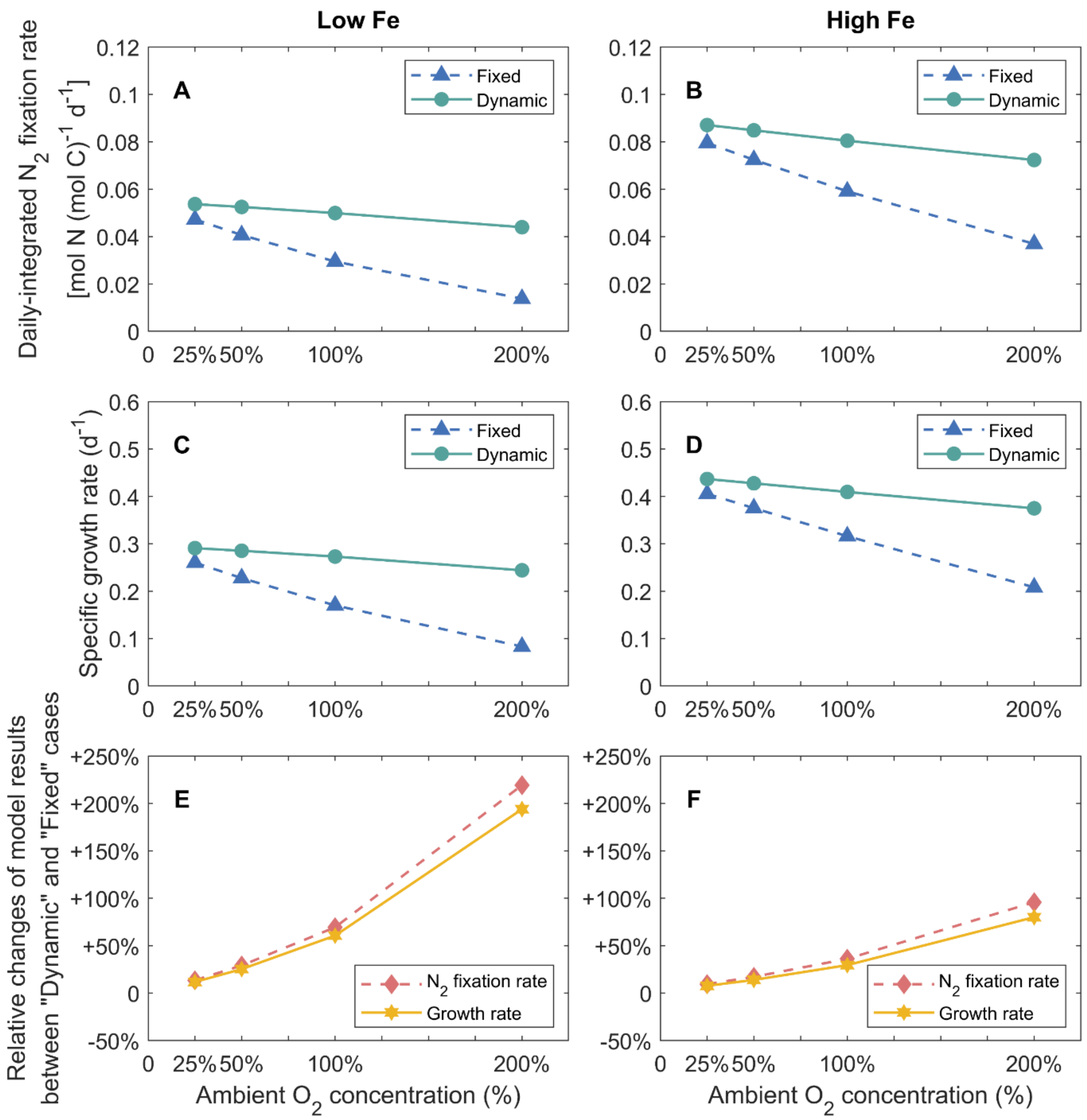

FIG S6 Simulated daily-integrated N_2 fixation rates (A, B), growth rates (C, D) and their relative changes (E, F) between "Dynamic permeability" and "Fixed permeability" cases under different ambient oxygen concentrations, from 25% to 200% of the saturation. The model simulations were performed under low-Fe (40 pM) and high-Fe (1250 pM) conditions.

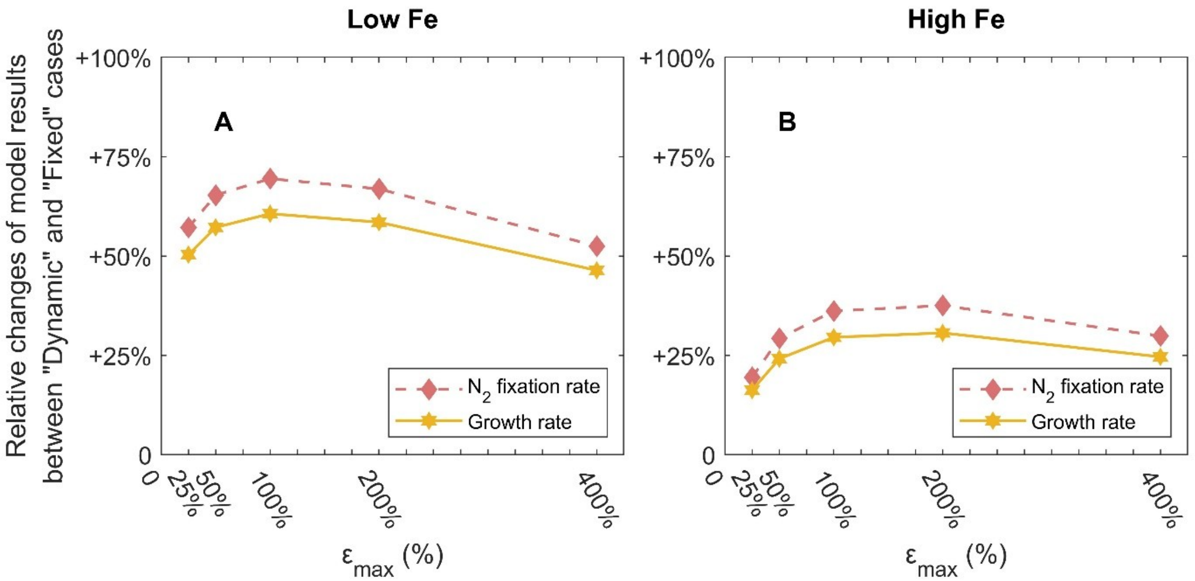

FIG S7 Simulated relative changes of daily-integrated N_2 fixation rates and growth rates between "Dynamic permeability" and "Fixed permeability" cases under different maximal relative diffusion coefficient (ε_{max}), from 25% to 400% of that in the standard case (Fig. 1B). The model simulations were performed under low-Fe (40 pM) and high-Fe (1250 pM) conditions.
